# *Xenopus* cell-free extracts and their applications in cell biology study

**DOI:** 10.52601/bpr.2023.230016

**Published:** 2023-08-31

**Authors:** Junjun Liu, Chuanmao Zhang

**Affiliations:** 1 Department of Biological Sciences, California State Polytechnic University, Pomona, CA 91768, USA; 2 The Academy for Cell and Life Health, Faculty of Life Science and Technology, Kunming University of Science and Technology, Kunming 650500, China; 3 The Key Laboratory of Cell Proliferation and Differentiation of the Ministry of Education, College of Life Sciences, Peking University, Beijing 100871, China

**Keywords:** *Xenopus*, Cell-free extract, Oocyte, Egg, Embryo, Meiosis, Mitosis, Spindle

## Abstract

*Xenopus* has proven to be a remarkably versatile model organism in the realm of biological research for numerous years, owing to its straightforward maintenance in laboratory settings and its abundant provision of ample-sized oocytes, eggs, and embryos. The cell cycle of these oocytes, eggs, and early embryos exhibits synchrony, and extracts derived from these cells serve various research purposes. Many fundamental concepts in biochemistry, cell biology, and development have been elucidated through the use of cell-free extracts derived from *Xenopus* cells. Over the past few decades, a wide array of cell-free extracts has been prepared from oocytes, eggs, and early embryos of different *Xenopus* species at varying cell cycle stages. Each of these extracts possesses distinct characteristics. This review provides a concise overview of the *Xenopus* species employed in laboratory research, the diverse types of cell-free extracts available, and their respective properties. Furthermore, this review delves into the extensive investigation of spindle assembly in *Xenopus* egg extracts, underscoring the versatility and potency of these cell-free systems in the realm of cell biology.

## INTRODUCTION

*Xenopus* was initially employed for research and educational purposes in South Africa for an extended period. Subsequently, its utility in human pregnancy tests was discovered (Elkan 1938). Due to its ease of laboratory maintenance, the simplicity of inducing spawning through hormone injections, and along with large-sized oocytes, eggs and embryos, *Xenopus* quickly gained popularity as a model animal system worldwide for the study of cell biology, molecular biology, and developmental biology, starting in the 1970s. Since then, numerous foundational concepts in cell biology and development have emerged from research using this system. These include insights into the molecular mechanisms of cell cycle regulation (Philpott and Yew 2008), the initial identification of crucial vertebrate developmental genes (De Robertis *et al.* 2000), and the elucidation of fundamental signaling pathways such as Wnt and BMP (bone morphogenetic protein) pathways (Bier and De Robertis 2015; Cruciat and Niehrs 2013).

Initially, *Xenopus* oocytes, eggs, and embryos were considered less suitable for live-cell imaging in cell biology studies due to their relatively low transparency. However, owing to their amenability to microinjection, high tolerance for light exposure, and the development of new imaging techniques, these intact cells have evolved into one of the most valuable models among vertebrates for cell biology research.

Nonetheless, a significant portion of cell biology research has been conducted using cytoplasmic extracts derived from *Xenopus* oocytes, eggs, and embryos. These cell-free systems offer several advantages. Firstly, proteins of interest can be selectively removed from the extracts through immunodepletion, and recombinant proteins or pharmaceutical agents can be easily introduced into the system. Secondly, this system yields a substantial quantity of material for biochemical analysis. For instance, a single female *Xenopus* *laevis* can produce several thousand eggs, with one egg estimated to contain roughly the same amount of total cellular proteins as approximately 10^6^ HeLa cells.

In this review, our focus will be on the species of *Xenopus* utilized in laboratory settings and the characteristics of extracts prepared from their oocytes, eggs, or embryos. Additionally, we will explore the applications of these extracts in the study of spindle assembly to underscore the versatility of these cell-free systems in advancing our understanding of cell biology.

## COMMONLY USED *XENOPUS* SPECIES IN LABORATORY

*Xenopus*, commonly referred to as the clawed frog, comprises a genus of highly aquatic frogs indigenous to sub-Saharan Africa, encompassing approximately twenty distinct species. Among these, three species — *Xenopus* *laevis* (African clawed frog), *Xenopus* *borealis* (Marsabit clawed frog), and *Xenopus* *tropicalis* (Western clawed frog) — have been adopted as experimental models. Most *Xenopus* species are tetraploid, with the notable exception of *Xenopus* *tropicalis*, which is diploid (Amaya *et al.* 1998; Harland and Grainger 2011).

The African clawed frog, *Xenopus* *laevis*, has played a pivotal role in advancing research within the fields of development and cell biology. It stands out as the largest of the three species, both at the organismal and cellular levels, and has been extensively employed for investigating facets of the cell cycle, DNA replication and repair, spindle assembly, and the functioning of microtubule motors. Despite the sequencing of its genome in 2016 (Session *et al.* 2016), the utility of this model organism for genetic and proteomic studies remains constrained due to its protracted generation time and tetraploidy.

*Xenopus* *borealis*, which is more closely related to *Xenopus* *laevis* than to *Xenopus* *tropicalis*, is a comparatively less utilized model organism. Until 1977, it was erroneously identified as *Xenopus* *mulleri* (Brown *et al.* 1977). In the 1970s, it was extensive used in comparative studies of ribosomal DNA (Brown and Sugimoto 1974; Brown *et al.* 1972; Griswold *et al.* 1974; Wellauer and Reeder 1975). It is slightly smaller than *Xenopus laevis*, resulting in correspondingly smaller eggs and spindles assembled in extracts derived from these eggs, compared to *Xenopus laevis*.

*Xenopus tropicalis*, the smallest of the three species, boasts a diploid genome and holds the distinction of being the first amphibian species to have its genome sequenced (Hellsten *et al.* 2010). Recently, it has gained traction as a research model in developmental genetics and functional genomics. Notably, microtubule regulatory factors in extracts from *Xenopus tropicalis* respond differently to stabilizing agents, such as chromosomes, yielding smaller microtubule structures. Consequently, *Xenopus tropicalis* eggs can be harnessed for the same assays previously established for *Xenopus laevis*, capitalizing on a richer array of molecular and genetic tools. However, due to their reduced size, extracts obtained from *Xenopus tropicalis* eggs are considerably more limited in quantity and are typically employed to explore intriguing cellular scaling phenomena, such as spindle size regulation.

## DIFFERENT TYPES OF *XENOPUS* CELL-FREE EXTRACTS

**Figure 1:**
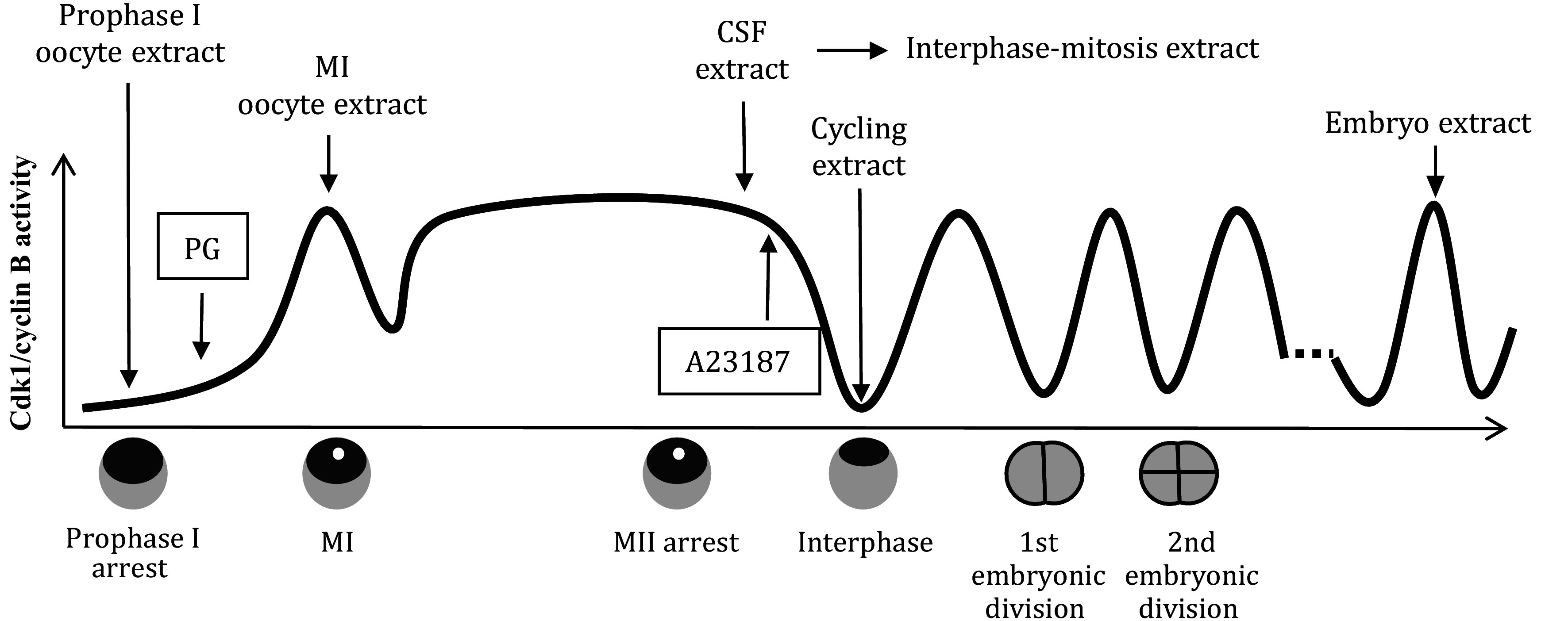
*Xenopus* cell-free extracts. Progesterone (PG) induces prophase I-arrested *Xenopus* oocyte to mature. At GVBD, white spots appear at the animal hemisphere of the maturing oocytes, and the mature oocytes arrest at metaphase II due to CSF, which is fertilizable. After fertilization, the CSF is released, and the mature eggs enter interphase and start synchronous embryonic cell division until blastula stage VIII when the synchrony is lost. In the laboratory, the fertilization is simulated by the addition of calcium ionophore A23187. Prophase I-arrested oocytes are used to prepare prophase I oocyte extracts. MI-arrested (by cold treatment) oocytes are used to prepare MI oocyte extracts. MII-arrested eggs are used to prepare CSF extracts, and interphase-mitosis extracts are prepared from the freshly prepared CSF extracts with the addition of calcium. Activated eggs are used to prepare cycling extracts. Early-stage embryos are used to prepare embryo extracts, which can be arrested in mitosis with the addition of cell cycle regulators

*Xenopus* oocytes, eggs, and early embryos offer a distinct synchronized system, allowing for the creation of cell-free extracts arrested at various stages of the cell cycle during the early development of *Xenopus* (refer to Fig. 1). In the following sections, we will provide a concise overview of the extraction process and delve into the distinctive attributes of these extracts.

### Oocyte extract

*Xenopus* oocytes, much like those of most mammals, experience their entire growth within the ovary and naturally reach a state of arrest at the prophase of meiosis I, irrespective of their oogenesis stages (I–VI) (Sato and Tokmakov 2020). These oocytes found within the ovaries are referred to as "immature" oocytes because they lack the capability for fertilization. In *Xenopus*, the hormone progesterone is considered a pivotal physiological factor that induces a process known as oocyte maturation. During this phase, fully developed Stage VI oocytes, initially classified as "immature" oocytes, exit the meiotic arrest, progress from prophase of MI (prophase I) to metaphase of MII (metaphase II), and then re-enter an arrest at metaphase II, thereby attaining fertilizability. Researchers have extensively investigated this process in intact oocytes, employing techniques like microinjection and biochemical analysis of cell lysates (Masui and Clarke 1979; Smith 1989). Nevertheless, the cytoplasmic extracts derived from these oocytes offer several experimental advantages, including their uniform composition and ease of biochemical manipulation, such as protein immunodepletion and the introduction of exogenous proteins or pharmacological agents.

In a study by Attardi *et al*., where *Xenopus* oocyte extracts were prepared for investigating DNA replication (Gandini Attardi *et al.* 1976), *Xenopus laevis* Stage VI oocyte nuclei and cytoplasm were initially collected separately. Nuclei and cytoplasmic extracts were subsequently obtained through centrifugation. However, contemporary oocyte extract preparation no longer involves the separation of nuclei and cytoplasm.

To prepare oocyte extracts today, oocytes are surgically removed from a female frog. Fully developed *Xenopus laevis* oocytes typically reach a diameter of 1.2 mm. A sexually mature *Xenopus* female harbors tens of thousands of oocytes at various stages of growth (Dumont 1972), thus providing access to thousands of matured oocytes (Stage VI). It is important to note that poor-quality oocytes from a single frog may undergo premature lysis, potentially triggering apoptosis in the entire extract and therefore should be removed before crushing the oocytes by centrifugal force. Typically, 3000–5000 oocytes yield approximately 500 µL of extract.

*Xenopus* oocyte extracts can be prepared from oocytes at two different cell cycle stages: prophase I and MI.

#### Prophase I oocyte extract

Prophase I-arrested Stage VI oocytes are obtained from *Xenopus* ovaries. These oocytes are carefully collected and tightly packed using silicone oil, such as Versilube F-50, before being subjected to high-speed centrifugation. From this process, cytoplasmic fractions, referred to as prophase I oocyte extracts, are collected from the centrifugation tubes. These extracts can either be used immediately or stored at –80°C. Though the frozen extracts can reproduce certain meiotic signaling (Shibuya *et al.* 1992), however, it is important to note that frozen extracts lose their protein synthesis capability, and therefore, studies requiring the *de novo* synthesis of signaling proteins should utilize freshly prepared oocyte extracts (Crane and Ruderman 2006).

Prophase I oocyte extracts are commonly employed for investigating signaling events following progesterone stimulation. Various stimuli, including progesterone (PG), protein kinase A inhibitor (PKI) (VanRenterghem *et al.* 1994), Mos protein (VanRenterghem *et al.* 1994), p42MAPK (ERK2) (Huang and Ferrell 1996), among others, have been used to trigger these signaling events. Following stimulation, samples of the extracts can be taken for immunoblotting and kinase assays.

Nevertheless, prophase I oocyte extracts have certain limitations. Firstly, cell cycle signaling often becomes activated spontaneously in these extracts, possibly due to a transient decrease in cyclin adenosine monophosphate (cAMP) levels and protein kinase A (PKA) activity, or the removal of inhibitors during extract preparation. The addition of a cAMP analog has been shown to inhibit this spontaneous activation (Qian *et al.* 2001). Secondly, it has been challenging to fully reproduce the complete signaling pathway from PG stimulation to Cdk1/cyclin B activation and reentry into meiosis in the extract. It is suspected that the separation of cytosolic and lipid fractions during extract preparation may disrupt membrane-associated signaling components. Despite these limitations, *Xenopus* oocyte extracts have nonetheless proven to be a potent system for investigating cellular regulatory events, particularly during meiosis.

#### MI oocyte extract

It is well-established that early meiotic progression in *Xenopus* oocytes exhibits asynchrony. The period from hormonal stimulation to the occurrence of germinal vesicle breakdown (GVBD) varies among oocytes, even those collected from the same frog at the same time (Ohsumi *et al.* 2006). Consequently, the preparation of extracts from prophase I-arrested Stage VI oocytes with a low degree of synchrony in meiotic progression has posed challenges for *in vitro* biochemical studies, particularly concerning the rapid transition from MI to MII. However, researchers have discovered that meiotic progression after GVBD is remarkably synchronous in maturing *Xenopus* oocytes (Ohsumi *et al.* 1994), and these maturing oocytes can be arrested at the MI stage (Iwabuchi *et al.* 2000). Building on these findings, Iwabuchi and colleagues developed cell-free extracts from MI-arrested oocytes, which recapitulate many of the cell cycle events during the MI to MII transition (Iwabuchi *et* *al.* 2000). These extracts are capable of mirroring the meiotic cell cycle of maturing oocytes, including the fluctuation of Cdk1/cyclin B activity, resembling that in maturing oocytes (Ohsumi *et al.* 2006).

The key to synchronizing oocytes at MI is cold treatment. After PG stimulation, GVBD oocytes are collected and incubated at 18 °C for 60 min followed by an additional incubation at 22 °C for 15 min. Ooctyes reaching MI during the incubation can be stored on ice until enough MI oocytes have been collected for extract preparation. The MI arrest by cold treatment is reversible if the treatment is less than 2 h. Once the oocytes are brought back to room temperature within 2 h of the onset of cold treatment, the oocytes resume meiosis and reach MII in the normal time schedule. The packing and crushing procedures of the oocytes are similar to that of prophase I oocyte extract preparation and are nicely described by Ohsumi *et al*. The progression of meiotic progression can be monitored by various methods including examining the morphological changes of sperm nuclei, histone H1 kinase assay, *etc*.

The key to synchronizing oocytes at MI is a cold treatment method. Following PG stimulation, GVBD oocytes are collected and incubated at 18 °C for 60 min, followed by an additional incubation at 22 °C for 15 min. Oocytes reaching MI during this incubation period can be stored on ice until a sufficient number of MI oocytes have been collected for extract preparation (Ohsumi *et al.* 2006). Importantly, MI arrest induced by cold treatment is reversible if the treatment lasts less than 2 h. If oocytes are returned to room temperature within 2 h of the onset of cold treatment, they resume meiosis and progress to MII according to the normal schedule. The procedures for packing and crushing the oocytes are similar to those used in prophase I oocyte extract preparation, as elegantly described by Ohsumi and colleagues (Ohsumi *et al.* 2006). The progression of meiotic development can be monitored through various methods, such as the examination of morphological changes in sperm nuclei and histone H1 kinase assays (Ohsumi *et al.* 2006).

### Egg extracts

Early *Xenopus* egg extracts were initially prepared as homogenates for *in vitro* investigations, including studies on DNA synthesis (Benbow and Ford 1975). Upon joining Maller's laboratory, Lohka, who had prior experience in preparing concentrated cytoplasmic extracts from *Rana pipiens* (Northern leopard frog) eggs (Lohka and Masui 1983a), collaborated with Maller to develop *Xenopus* egg extracts arrested at the MII stage of meiosis. This was done to study the maturation-promoting factor (MPF) (Lohka and Maller 1985). Instead of homogenization, they employed a method of crushing densely packed eggs using centrifugal force, resulting in highly concentrated cytoplasm. *Xenopus* egg extracts prepared through this innovative approach were kept in a crude and concentrated form, closely mimicking *in vivo* conditions and retaining many properties of cellular processes. Their work, as well as subsequent research by others, has introduced a novel and potent approach to studying complex and dynamic cellular processes *in vitro* (Lohka and Maller 1985; Murray 1991; Murray and Kirschner 1989; Sawin and Mitchison 1991).

*Xenopus* egg extracts can be obtained from eggs that are naturally arrested at MII or from parthenogenetically activated eggs that have been released from the MII arrest. Depending on the cell cycle stage of the egg extracts, they can be conveniently employed to investigate various aspects of cell cycle events, such as DNA replication, nuclear envelope formation, chromosome condensation, and kinetochore formation. Later in this review, we will utilize the study of spindle assembly as an example to demonstrate the versatility and effectiveness of these extracts in cell biology research.

#### CSF extracts

The initial *Xenopus* egg extract created by Lohka and Maller was derived from eggs arrested at the MII stage. This arrest is induced by a cytostatic factor, known as CSF (Masui and Markert 1971), and consequently, the extract is often referred to as CSF extract. This extract effectively maintains the metaphase arrest and has been extensively utilized for studying spindle assembly, achieved through the introduction of sperm nuclei or chromatin-coated beads (Heald *et al.* 1996; Sawin and Mitchison 1991). Researchers have the option to use these extracts either immediately after preparation or employ a modified method that allows for freezing, while still preserving spindle assembly activity at a level comparable to that of freshly prepared extracts.

#### Fresh CSF extracts

**Figure 2:**
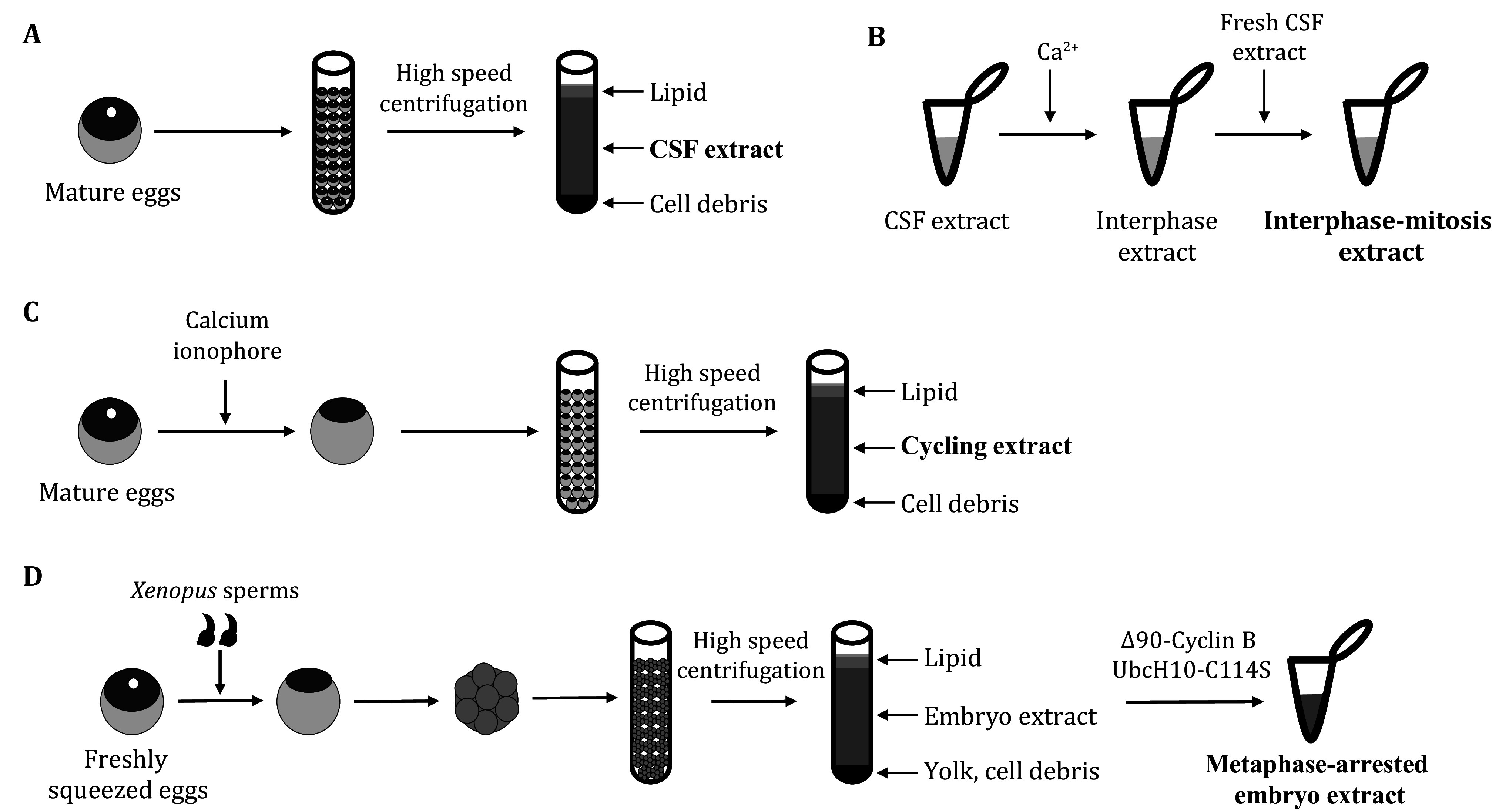
*Xenopus* egg/embryo extracts. **A** MII-arrested *Xenopus* eggs are crushed by high-speed centrifugation to make CSF extract. **B** The CSF extract is added with calcium to release CSF arrest to allow the extract to enter interphase. With the addition of freshly prepared CSF extract, the system is driven back to and arrest at mitosis, and the extract is sometimes called interphase-mitosis extract. **C** MII-arrested *Xenopus* eggs are released to interphase with the addition of calcium ionophore A23187. Forty-five minutes after the calcium ionophore addition, the CSF-released eggs are used to prepare the cycling extract. **D** Mature *Xenopus* eggs are fertilized by sperm slurry prepared from *Xenopus* males and early embryonic development starts. These early *Xenopus* embryos at different development stages, *e*.*g*. Stage IV or Stage VIII, are used to prepare embryo extract. These extracts are added with cell cycle regulators such as Δ90-cyclin B and UbcH10-C114S to arrest the extracts at mitosis

The procedure for preparing fresh CSF extract from *Xenopus laevis* was originally outlined by Lohka and Maller (Lohka and Maller 1985), and subsequently by other researchers (Murray 1991). Typically, 3–4 female *Xenopus laevis* frogs are employed for each extract preparation. Each female frog is primed by injecting pregnant mare serum gonadotropin (PMSG) 48–72 h prior to the experiment. In the evening preceding the experiment, the animals are injected with human chorionic gonadotropin (hCG). After 12–14 h, the laid eggs are collected, de-jellied, and washed. The de-jellied eggs are then transferred to centrifuge tubes and subjected to centrifugal force for crushing. The cytoplasmic material located between the lipid cap and the yolk in the pellet is carefully transferred to another centrifuge tube, with the addition of cytochalasin D to inhibit actin polymerization. Following another round of centrifugation, the resulting supernatant constitutes the CSF extract, which is now ready for downstream experiments (Fig. 2A).

*Xenopus tropicalis*, in comparison to *Xenopus laevis*, exhibits differences in body size, timing of egg laying, and higher physiological temperature. Furthermore, their eggs are more sensitive to salt. Consequently, the procedure for preparing CSF extract using eggs from *Xenopus tropicalis* has been appropriately modified. For instance, due to their smaller size, eggs obtained from each female frog may yield only 0.1–0.2 mL of extract. Hence, it is common to use a larger number of *Xenopus tropicalis* females, typically 6–8, for the preparation of approximately 1 mL of extract. Detailed protocols for preparing *Xenopus tropicalis* egg extracts can be found elsewhere (Good and Heald 2018).

In addition to the aforementioned two egg extracts, egg extracts derived from *Xenopus borealis* were employed as a third *in vitro* system to investigate spindle interspecies scaling and morphometric variation (Kitaoka *et al.* 2018). MII-arrested *Xenopus borealis* egg extracts were prepared in a similar manner to *Xenopus laevis* egg extracts, with some modifications that were meticulously described by Kitaoka and colleagues (Kitaoka *et al.* 2018). It was observed that the spindles formed in *Xenopus borealis* egg extracts exhibited similar characteristics to those formed in the egg extracts of the other two species. However, there was a notable difference in the distribution of microtubules along the length of the *Xenopus borealis* spindles (Kitaoka *et al.* 2018).

#### Frozen CSF extracsts

Freshly prepared egg extracts often exhibit variations in quality from batch to batch, primarily influenced by factors such as egg quality and the intricacies of the preparation process. These variations frequently result in significant disparities in experimental outcomes, such as spindle size and shape (Grenfell *et al.* 2016; Hannak and Heald 2006), making it challenging to obtain reproducible data or gather a substantial number of datasets under controlled biological conditions. One potential solution is to produce a substantial quantity of egg extracts and store them in aliquots in a freezer. However, freshly prepared CSF extract has limited storage stability. While frozen extracts are frequently employed in laboratories to reconstitute essential cell cycle events like DNA replication and chromosome organization, their degradation can impair their capacity for critical cellular processes, such as *in vitro* spindle assembly. To tackle this issue, Takagi and colleagues devised a straightforward two-step method for preparing frozen egg extracts that maintain spindle assembly activity levels akin to those of freshly prepared extracts (Takagi and Shimamoto 2017).

To prevent the formation of ice crystals within the cytoplasm during cryopreservation, a phenomenon that can cause substantial damage to organelles and other cellular structures (Fowler and Toner 2005), Takagi and colleagues implemented a procedure involving the centrifugation of freshly prepared CSF extracts through a filter device with a 100-kDa membrane. This separation process yielded two distinct fractions: the extract concentrates and the flow-through. These two fractions were separately frozen and stored. Before usage, these two fractions were thawed on ice and then combined to reconstitute an extract resembling fresh CSF extract. With this innovative approach, the authors were able to amass extensive data when studying the correlations between metaphase spindle size and stiffness (Takagi and Shimamoto 2017).

#### Interphase-mitosis extract

To initiate the assembly of spindles *in vitro*, sperm nuclei are introduced into the CSF extract. As each sperm nucleus is paired with a centriole, incubation within the extract prompts the formation of a half spindle around the sperm nucleus. Over time, this half spindle merges with another half spindle, ultimately creating a bipolar spindle structure. When the CSF extract is of high quality, spindles can even form without the presence of half spindles or asters (Good 2016; Liu *et al.* 2004). However, it's important to note that while sufficient for certain applications, these spindles are less physiologically relevant and lack functional kinetochores.

To tackle this limitation, the CSF extract can be transitioned from a CSF-arrested state to an interphase by introducing calcium (Lohka and Maller 1985). This transition allows for the replication of sperm nuclei DNA and centrosomes. Upon re-entry into mitosis, achieved by either adding an equal amount of fresh CSF extract (Sawin and Mitchison 1991) or spontaneous re-entry in high-quality CSF extract (Liu and Maller 2005), bipolar spindles form in a more physiologically relevant manner. This modified extract is commonly referred to as an “interphase-to-mitotic extract” or “interphase-mitosis extract” (Fig. 2B) (Sawin and Mitchison 1991), and it facilitates the assembly of a complete spindle with guidance from a single sperm nucleus within this extract.

#### Cycling extract

*Xenopus* egg extracts can also be obtained from parthenogenetically activated eggs, achieved through either electrical shock or treatment with the calcium ionophore A23187 (Murray 1991; Murray and Kirschner 1989; Tunquist *et al.* 2002). These extracts are typically prepared 45 min after exiting the MII stage. Due to their capability to undergo multiple rounds of DNA replication and mitosis *in vitro*, they are commonly referred to as “cycling extracts”, which are proficient at assembling bipolar spindles (Fig. 2C) (Tunquist *et al.* 2002). The pioneering work of Lohka and Masui involved the creation of the first extract of this kind by activating Rana pipians eggs and subsequently subjecting them to centrifugal force for extraction (Lohka and Masui 1983a). Subsequently, other researchers developed extracts with the ability to undergo multiple cell cycles *in vitro* (Hutchison *et al.* 1987; Murray and Kirschner 1989). Freshly prepared cycling extracts can indeed undergo multiple cell cycles, while frozen extracts are generally limited to a single cell cycle (Murray 1991). To initiate spindle assembly, demembranated sperm nuclei are introduced into the extract, and the cell cycle is initiated by warming up the extract to 22 °C (Tunquist *et al.* 2002).

### Embryo extracts

Another type of *Xenopus* cytoplasmic extract is derived from *Xenopus laevis* embryos. The cell cycle of *Xenopus laevis* embryonic cells is synchronized up to blastula stage VIII, allowing for the preparation of extracts arrested in mitosis from these synchronized embryonic cells (Fig. 2D). The metaphase arrest can be further fortified by introducing regulators of the cell cycle machinery, such as non-degradable cyclin B delta 90 (Glotzer *et al.* 1991), and can be further intensified with the addition of a dominant negative inhibitor of the anaphase promoting complex/cyclosome (APC/C), such as UbcH10-C114S (Townsley *et al.* 1997). It's important to note that embryo extracts are obtained in considerably smaller quantities compared to egg extracts, and their cell cycles are not as precisely synchronized. Additionally, embryo extracts are less robust than egg extracts and are primarily utilized to investigate the molecular mechanisms governing spindle size regulation (Good and Heald 2018; Wilbur and Heald 2013; Wuhr *et al.* 2008).

Because the amount of embryo extract that can be obtained is significantly smaller than that from eggs, *Xenopus* *tropicalis* embryos are not suitable for this type of preparation (Good and Heald 2018). It's preferable to use eggs extracted from female frogs, as opposed to laid eggs, for fertilization and embryo development. It is also essential to empirically determine the optimal time point for crushing embryos at each developmental stage to generate the most functional embryo extracts (Wilbur and Heald 2013). High-quality embryo extracts have been successfully prepared from Stages IV and VIII embryos (Good 2016). Detailed preparation procedures have been elegantly elucidated by Good and Heald (Good and Heald 2018). Furthermore, interphase *Xenopus* embryo extracts can also be prepared for the study of nuclear scaling (Levy and Heald 2010).

## INVESTIGATION OF SPINDLE ASSEMBLY IN *XENOPUS* CELL-FREE EXTRACTS

### Spindle assembly in *Xenopus* cell-free extracts

The mitotic spindle plays a pivotal role in ensuring the accurate segregation of chromosomes into the two daughter cells, thus preserving genome integrity. The assembly of the spindle is an intricately complex process involving hundreds of proteins. Understanding the regulation of this process has been a central focus in cell biology for decades. However, research in this area has faced challenges due to the intricate cellular environment and the cell-cycle checkpoint machinery that tightly controls spindle assembly.

*Xenopus* cell-free extracts have emerged as a potent cell cycle system for investigating spindle assembly *in vitro*. These extracts offer many advantages, including the ability to manipulate the spindle assembly process biochemically without disrupting the spindle assembly checkpoint. Additionally, they provide a readily available and abundant supply of materials for thorough biochemical analysis.

A variety of DNA templates, ranging from single and double-stranded plasmids (Blow and Laskey 1986) to mammalian nuclei (Leno *et al.* 1992), can be replicated within *Xenopus laevis* egg extracts, leading to spindle assembly. However, for these egg/embryo extracts, *Xenopus laevis* sperm nuclei are the preferred and physiologically relevant substrates. The method for preparing sperm nuclei was initially outlined by Lohka and Masui (Lohka and Masui 1983a, b) and has since been detailed by other researchers (Gillespie *et al.* 2012; Hazel and Gatlin 2018; Miyamoto *et al.* 2015). Each demembranated sperm nucleus retains a basal body containing two centrioles, which can serve as the nucleation sites for microtubules within the extract (Blachon *et al.* 2014; Felix *et al.* 1994). The prepared sperm nuclei can be stored in aliquots at –80 °C until they are ready for use.

**Figure 3:**
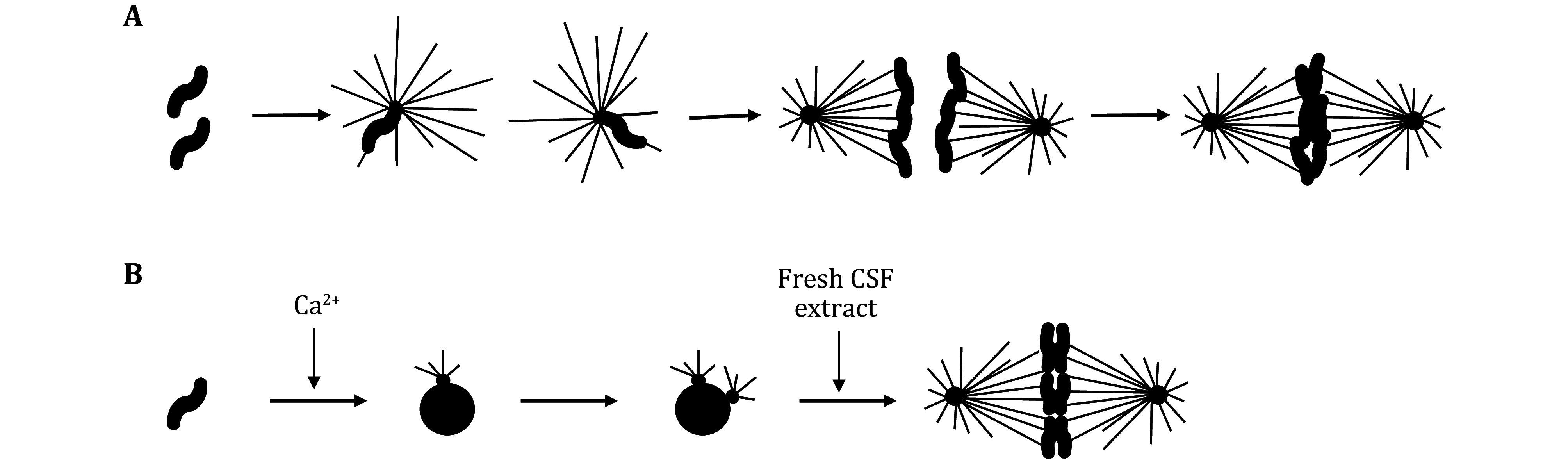
Two pathways to spindle assembly *in vitro*. **A** In *Xenopus* CSF extract, each sperm nucleus contains one centrosome and directs the assembly of the one-half spindle. Two half spindles then fuse and form a bipolar spindle. **B** In extracts that go through interphase, the centrosome and DNA of the sperm nuclei will replicate, and each sperm nucleus is capable of the assembly of a bipolar spindle in this case

In CSF extracts, the frozen sperm nuclei, once thawed, are introduced into the extracts to initiate spindle assembly. Since each demembranated sperm nucleus retains a single centrosome, it facilitates the formation of a half spindle within the CSF extract. These half spindles can then merge to form bipolar spindles (Sawin and Mitchison 1991; Wignall and Heald 2001) (Fig. 3A). Additionally, sperm nuclei can be employed to generate interphase sperm nuclei (Good *et al.* 2013). To achieve this, after adding sperm nuclei, the CSF extract is transitioned to the interphase state by introducing calcium, resulting in the formation of interphase nuclei. These interphase nuclei can be isolated and subsequently added to egg extract to facilitate spindle assembly. Given that interphase nuclei have undergone DNA and centrosome replication, they effectively support the formation of bipolar spindles when introduced into CSF extract (Sawin and Mitchison 1991; Wignall and Heald 2001) (Fig. 3B).

In the interphase-mitosis extract, the spindle assembly is achieved by either adding the sperm nuclei to the CSF extract before the introduction of calcium or by adding them concurrently with calcium, which prompts the extract to transition to interphase (Field and Mitchison 2018; Sawin and Mitchison 1991). The release from CSF arrest triggers the formation of nuclear envelopes around each sperm nucleus, along with DNA replication and centrosome duplication. Upon re-entry into mitosis, a single sperm nucleus has the capacity to orchestrate the assembly of a complete spindle, facilitated by the duplication of centrosomes during interphase. These duplicated centrosomes then separate, leading to the formation of bipolar spindles, a phenomenon often referred to as "cycled CSF spindles" (Field and Mitchison 2018).

The cycling extract is also employed to investigate spindle assembly *in vitro*. Tunquist and colleagues demonstrated that by supplementing the cycling extract with the cyclin E/Cdk2 AF mutant, the addition of demembranated sperm nuclei, and subsequently initiating the cycling process by warming the extract to 22 °C, the extract can be arrested in the subsequent metaphase, resulting in the formation of functional mitotic bipolar spindles (Tunquist *et al.* 2002).

To investigate centrosome-independent spindle assembly, DNA materials devoid of centrosomes can be introduced into the extracts. Typically, plasmid DNA is tethered to either glass or magnetic beads, facilitating their assembly into chromatin structures within the extracts. These synthetic chromatins then stimulate microtubule polymerization and promote spindle assembly (Heald *et al.* 1996).

*Xenopus* embryo extract enables the assembly of mitotic spindles rather than meiotic ones. The size of the spindles formed in this extract correlates with the developmental stages of the embryos from which the extract is derived (Wilbur and Heald 2013). Since *Xenopus* embryos lack the CSF activity, which is crucial for synchronized mitotic arrest during the spindle assembly process, specific measures are taken. These measures involve the addition of a non-degradable form of cyclin B (Δ90-CyclinB1) (Glotzer *et al.* 1991) and a dominant-negative variant of the ubiquitinating enzyme UbcH10 (UbcH10-C114s). This latter component functions as a dominant negative inhibitor of the anaphase promoting complex (APC) (Townsley *et al.* 1997). In some cases, a 5%–10% CSF extract is supplemented with the embryo extract to further enhance metaphase arrest and improve spindle formation activity (Good 2016; Good and Heald 2018).

#### Regulation of spindle size in vitro

The size of mitotic spindles is known to exhibit a positive correlation with cell size. Spindle length, which is defined as the distance between two spindle poles, is tightly regulated during metaphase to ensure an adequate distance for the segregation of duplicated chromosomes into daughter cells. *Xenopus* egg extracts have been instrumental in the study of spindle size. Through the utilization of microfluidic technology, Good and colleagues established an encapsulated system containing extracts from *Xenopus* eggs or embryos. This system demonstrated that the size of mitotic spindles exhibits a near-linear correlation with the volume of cytoplasmic materials, rather than their shape (Good *et al.* 2013). It was revealed that altering the cytoplasmic volume alone is sufficient to modulate spindle length, replicating the scaling phenomena observed in *Xenopus* cells during development (Good *et al.* 2013; Hazel *et al.* 2013).

Conversely, when 400% more DNA in the form of chromatin-coated beads was introduced into *Xenopus* egg extract, the resultant spindle size exhibited only a marginal 10% increase (Dinarina *et al.* 2009). Additionally, the size of chromosomes in *Xenopus* *tropicalis* is approximately half that of *Xenopus laevis*. However, when *Xenopus tropicalis* chromosomes were introduced into *Xenopus laevis* extract, the spindles formed were only 10% shorter in length (Brown *et al.* 2007). These findings suggest a correlation between genome size and spindle size, though it is not linear, and the influence of genome size on spindle dimensions appears to be limited.

## OTHER APPLICATIONS OF *XENOPUS* CELL-FREE EXTRACTS

Spindle assembly serves as an excellent example, demonstrating the versatility of *Xenopus* cell-free extracts. In addition to spindle assembly, these extracts have been widely employed in the study of various cell cycle processes. A recent discovery has shown that when demembranated sperm nuclei are added, cell-like compartments form 23 min after the initiation of the cell cycle. Remarkably, well-defined mitotic spindles are assembled in *Xenopus laevis* egg extracts just 10 min later (Cheng and Ferrell 2019). These cell-like compartments are not only capable of replicative division but also give rise to two daughter compartments at the end of the mitotic cycle. It's worth noting that these cell-like compartments can be formed independently of nuclei, centrosomes, or microfilaments, but they do require microtubule polymerization (Cheng and Ferrell 2019).

*Xenopus laevis* egg extracts are rich in components that facilitate the *in vitro* formation of an endoplasmic reticulum (ER) network. This ER network can be assembled in either interphase- or metaphase-arrested *Xenopus* egg extracts by introducing purified microsomes (Wang *et al.* 2013*,* 2019). Significantly, the resulting network closely resembles the one observed in cultured cells.

In addition to the aforementioned applications, *Xenopus* cell-free extracts have been instrumental in studying various cellular events, including DNA replication (Blow and Laskey 1986), chromosome condensation (Lohka and Maller 1985), kinetochore formation (Lohka and Maller 1985), and nucleus formation (Chen and Levy 2018), among others.

## SUMMARY

This review provides a summary of the preparation and characteristics of different types of extracts that have been employed in cell biology studies. Cell-free cytoplasmic extracts can be derived from various stages of *Xenopus* oocytes and embryos, including prophase I and MI-arrested oocytes, MII-arrested eggs, activated eggs, and M phase-arrested embryos. These extracts are characterized by their high concentration, closely mimicking *in vivo* conditions. They are amenable to precise biochemical manipulation, allowing for the removal of specific proteins of interest and the addition of recombinant proteins to the system. Furthermore, these systems offer an abundant supply of materials for comprehensive biochemical analyses. Given their numerous advantages, *Xenopus* cell-free extracts are poised to remain an invaluable and powerful tool in the field of cell biology research.

## Conflict of interest

Junjun Liu and Chuanmao Zhang declare that they have no conflict of interest.
